# The Self-Assembling Peptide P_11_-4 Induces the Expression of Mineralization-Related Genes in Odontoblasts Independently of Metabolic Alterations

**DOI:** 10.3390/jfb17010050

**Published:** 2026-01-18

**Authors:** Leticia Martins Pereira, Marina Damasceno e Souza de Carvalho Chiari, Diego Mauro Carneiro Pereira, Regina Maria Puppin-Rontani, Fábio Dupart Nascimento

**Affiliations:** 1 Departamento de Saúde Coletiva, Odontopediatria e Ortodontia, Pediatric Dentistry Division, Faculdade de Odontologia de Piracicaba, Universidade Estadual de Campinas, Av. Limeira, 901—Areião, Piracicaba 13414-903, SP, Brazil; L229497@dac.unicamp.br; 2 Dental Research Division, Paulista University, São Paulo 04026-002, SP, Brazil; marina.chiari@unifesp.br; 3 Department of Biochemistry, Molecular Biology Division, Federal University of São Paulo—UNIFESP/EPM, Rua Três de Maio, 100, São Paulo 04044-020, SP, Brazil; diego.mauro@unifesp.br

**Keywords:** self-assembling peptide, odontoblast-like cells, biomineralization

## Abstract

(1) Background: The synthetic eleven-amino acid peptide P_11_-4, derived from DMP-1, self-assembles into β-sheet tapes, ribbons, fibrils, and fibers that form a 3D matrix enriched with calcium-binding sites. This study investigated whether P_11_-4 modulates gene and protein expression or induces adverse metabolic alterations in odontoblast-like cells. (2) Methods: MDPC-23 cells were cultured under standard conditions and stimulated with different concentrations of P_11_-4, followed by assessments of cell viability using the MTT assay, proliferation and migration, cytoplasmic calcium kinetics, reactive oxygen species (ROS) production, osteogenic differentiation-related gene expression via PCR array, and expression of the pro-inflammatory cytokine interleukin-6 (IL-6) using confocal microscopy and flow cytometry. (3) Results: The MTT assay showed that P_11_-4 at 6.3, 12.6, and 25.2 µmol/L was non-cytotoxic and did not alter MDPC-23 cell proliferation or migration. Only the 25.2 µmol/L concentration induced a detectable Ca^2+^ influx and a slight increase in ROS. Among the 84 genes examined, P_11_-4 at 6.3 µmol/L upregulated 79 genes, including transcription factors, signaling molecules, and extracellular matrix-related proteins. Furthermore, P_11_-4 did not increase IL-6 expression under any condition tested. (4) Conclusion: P_11_-4 markedly modulates mineralization-associated gene regulation without causing metabolic damage in odontoblast-like cells.

## 1. Introduction

In contemporary dentistry, there is a growing interest in the study of biomimetic materials that can actively interact with dental tissues, promoting not only improved dentin-restoration adhesion but also the biological repair of affected structures [1]. In this context, the search for dental alternatives that facilitate biologically active dentin repair has intensified, driven by advancements in regenerative processes and the need for less invasive therapies.

Several biomimetic strategies have been developed to achieve collagen mineralization, drawing inspiration from the behavior of the protein matrix in natural biomineralization processes. These strategies are designed based on the amphiphilic properties of non-collagenous proteins (NCPs) such as DMP1 and dentin sialophosphoprotein (DSPP). Many of these macromolecules contain amino acids like aspartic and glutamic acid, phosphorylated serine, and threonine, which are thought to play a crucial role in mineral deposition due to their strong affinity for calcium ions (Ca^2+^) and collagen. In this regard, recent studies have demonstrated the mineralizing capacity of P_11_-4 [2,3], a synthetic eleven-amino acid-long peptide with a sequence that self-assembles into a 3D matrix with multiple calcium-binding sites derived from DMP-1. This peptide exhibits an amphiphilic nature that enables it to transition from a Newtonian fluid to a nematic gel characterized by organized domains. It undergoes hierarchical self-assembly to form β-sheet tapes, ribbons, fibrils, and fibers [4]. In water at pH levels above 7.5, it exists as monomers in random coil conformations; however, at low pH, it adopts an antiparallel β-sheet conformation. Additionally, it self-assembles under physiological conditions in a concentration-dependent manner [1,5]. More, P_11_-4 is related to cell adhesion and differentiation, as well as enhancing persistent tissue repair.

Since P_11_-4 can induce significant structural changes in both the mineral and organic components of dentin [6], investigating the cellular mechanisms that govern these processes using an odontoblast-like cell model will address a topic that has not been explored by previous studies in this specific field. This research approach will help elucidate the mode of action of P_11_-4 and the potential biological effects of its interaction on cell metabolism.

Our null hypothesis is that the P_11_-4 peptide: (1) cannot trigger cell damage, death, or interfere with cell migration and inflammatory responses; (2) cannot modulate the expression of genes and proteins that promote dentin matrix mineralization.

## 2. Materials and Methods

### 2.1. Cell Culture

MDPC-23, odontoblast-like cells were obtained from Nascimento’s lab (Dr. Fábio D. Nascimento), and cultured in Dulbecco’s Modified Eagle Medium (D-MEM, Life Technologies, Grand Island, NY, USA) supplemented with 10% fetal bovine serum (FBS) and 1.0% penicillin-streptomycin (P/S) (GIBCO^TM^, Life Technologies), previously filtered through a 0.22 µm membrane filter (Gibco, Grand Island, NY, USA). The cultures were maintained at 37 °C in a humidified atmosphere with 5% CO_2_.

### 2.2. Cell Viability Assay

Cell viability in the presence of P_11_-4 was evaluated using the reduction of 3-(4,5-dimethylthiazol-2-yl)-2,5-diphenyltetrazolium bromide (MTT), which forms formazan crystals through the action of mitochondrial reductases. The MTT assay kit (CellTiter Non-Radioactive Cell Proliferation Assay, Promega, Madison, WI, USA) was used in 96-well plates. Initially, 3 × 10^4^ MDPC-23 cells per well were seeded and incubated for 24, 48, and 72 h at 37 °C in a 5% CO_2_ atmosphere in a serial dilution of P_11_-4, 10, 20, or 40 µg/mL (6.3, 12.6, and 25.2 µmol/L). All experimental conditions were performed in triplicate for each group. Subsequently, 50 µL of MTT solution was added to each well containing the cells. After incubation, the MTT solution was aspirated, and the resulting formazan crystals were dissolved in 100 µL of DMSO. Once complete dissolution was achieved, the plates were transferred to a microplate reader (VersaMax Molecular Devices, CA, USA). Absorbance was measured at a wavelength of 570 nm, and cell viability values were expressed as the mean ± standard deviation.

### 2.3. Cell Proliferation Assay

MDPC-23 cells were cultured at a density of 1 × 10^4^ cells per well in 24-well plates until reaching 60–70% confluence. Once this confluence was achieved, FBS was withdrawn for 24 h to synchronize the cell cycle. Subsequently, cells were incubated with the peptide at concentrations of 6.3, 12.6, and 25.2 µmol/L in serum-free medium for 6 h at 37 °C. After this 6-h incubation, 10% of fetal bovine serum was added to the medium, and cells were further incubated for 72 h. After this period, cells were washed with PBS, fixed with methanol for 20 min at room temperature, and washed again with PBS. Finally, DAPI (4′,6-diamidino-2-phenylindole) (Molecular Probes, Eugene, OR, USA) was diluted 1:100,000 in PBS, and it was added and incubated for 20–30 min in the dark at room temperature. After incubation, each well was washed three times with PBS, and the plates were read using the InCell Analyzer 2200 (Cytiva, Marlborough, MA, USA). All experimental conditions were performed in triplicate for each group. Images were subsequently analyzed with InCell Analyzer Workstation 3.7.3, and results were expressed as the mean ± standard deviation.

### 2.4. Cell Migration Assay

MDPC-23 cells (3.0 × 10^4^) were seeded in 24-well plates and cultured in a humidified incubator at 37 °C with 2.5% CO_2_ until reaching confluence. To inhibit cell proliferation and ensure that subsequent cell behavior resulted primarily from migration, the cells were pre-treated with mitomycin C (10 µg/mL) diluted in D-MEM without FBS for 1 h. Following treatment, the wells were washed twice with serum-free medium, and the cells were exposed to increasing concentrations of the P_11_-4 peptide (6.3, 12.6, and 25.2 µmol/L) for the entire experimental period. A linear scratch was then created in the center of each well to define the migration area. All experimental conditions were performed in triplicate for each group. Images were captured at 12, 24, and 72 h using an inverted microscope equipped with a digital camera Axiocam 208 Color (Carl Zeiss, Oberkochen, Germany), acquiring two representative fields per well. One representative field per group for each time point was selected for presentation in the Results section.

### 2.5. Kinetic Evaluation of Cytoplasmic Calcium Concentration

The variation in cytoplasmic Ca^2+^ concentration in MDPC-23 cells in response to the P_11_-4 peptide was monitored using the Victor Nivo system (Revvity, Waltham, MA, USA, USA). Cells were cultured at a density of 5 × 10^4^ cells per well in black-walled, clear-bottom 96-well plates. The cells were maintained in culture for 48 h prior to the experiments. Before measuring calcium influx (Ca^2+^), the culture medium was carefully removed and replaced with 50 µL per well of Fluo-4 NW Calcium Assay Kit reagent (ThermoFischer Scientific, Walthan, MA, USA), according to the manufacturer’s instructions. Cells were incubated for 60 min at 37 °C, protected from light. Subsequently, the plate was placed in the fluorimeter, and samples were excited at a wavelength of 490 nm, with fluorescence emission detected at 530 nm. Samples were monitored at 37 °C, and fluorescence was recorded at 3-s intervals over 200 s, totaling 65 readings per well. After an initial 30-s monitoring period to measure basal fluorescence intensity of unstimulated cells, the cells were stimulated with 6.3, 12.6, and 25.2 µmol/L of the P_11_-4 peptide, which was automatically injected by the instrument to achieve predetermined final concentrations. The intracellular calcium transients ([Ca^2+^]i) were then monitored for the following 180 s. Fluorescence readings were exported to an Excel (Microsoft, Washington, DC, USA) file and calculated as the maximum fluorescence obtained after peptide addition minus the basal fluorescence intensity. Experiments were performed in triplicate, and results were analyzed using GraphPad Prism 4 software (Dotmatics, Boston, MA, USA) and presented graphically as the mean fluorescence values.

### 2.6. Reactive Oxygen Species (ROS) Production Assay

The production of ROS in MDPC-23 cells was determined using the fluorogenic probe H_2_DCFDA (Life Technologies, Carlsbad, CA, USA). Fluorescence was monitored by confocal microscopy using a Leica SP8 microscope (Leica Microsystems, Wetzlar, Germany). Cells were cultured in 35 mm glass-bottom culture plates at a density of 1 × 10^5^ cells/well for 48 h and subsequently incubated with 6.3, 12.6, and 25.2 µmol/L of the P_11_-4 peptide or hydrogen peroxide (100 µmol/L) as a positive control, using a transwell system for 4 h, to assess ROS levels triggered by the peptide. After incubation, cells were washed with PBS and incubated with 5 µmol/L of H_2_DCFDA (0.5% DMSO) in Tyrode’s solution for 30 min at 37 °C. All experimental conditions were performed in triplicate for each group. Following incubation, the cells were washed with culture medium and maintained in Tyrode’s solution for imaging. Image acquisition and analysis were performed using LAS X software (Version 5.3.1, Leica Microsystems). Representative images from each experimental group were selected for data presentation.

### 2.7. Osteogenic Differentiation-Related Gene Expression Analysis Using the Osteogenesis RT2 Profiler^TM^ PCR Array

The osteogenesis RT2 Profiler™ PCR kit (QIAGEN, Hilden, Germany) enabled the evaluation of 96 genes associated with osteogenic differentiation. For this purpose, cells were plated, and upon reaching 80% confluence, the culture medium was replaced with D-MEM FBS for a period of 24 h. After this period, the cells were incubated for 6 h with 6.3 µmol/L of P_11_-4 peptide. Immediately afterward, total RNA was extracted using the RNeasy Mini Kit, and cDNA was synthesized using the RT^2^ First Strand Kit (both QIAGEN). CT values were exported to an Excel file to create a CT value table. This table was then uploaded to the data analysis web portal at http://www.qiagen.com/geneglobe (accessed on 28 February 2025). Samples were assigned to control and test groups. CT values were normalized based on an automatic selection from a complete panel of reference genes.

To evaluate the pathways and processes associated with the analyzed genes, an over-representation analysis was performed using the clusterProfiler package [7,8]. Enrichment analyses of Kyoto Encyclopedia of Genes and Genomes (KEGG) pathways and Gene Ontology cellular components were conducted. It was considered significant when FDR < 0.05.

### 2.8. Interleukin-6 Expression Evaluation by Flow Cytometry

Approximately 1 × 10^5^ cells were cultured in culture plates until reaching 80% confluence and incubated with 6.3, 12.6, and 25.2 µmol/L of the P_11_-4 peptide for 4 h. To initiate the experiment, the culture medium was discarded, and the adherent cells were detached from the plates using 10 mmol/L ethylenediaminetetraacetic acid (EDTA) solution in PBS buffer. The cell suspension was collected in tubes, centrifuged at 1500 rpm for 3 min, the supernatant discarded, and the cells were washed twice with PBS buffer. After another centrifugation, the supernatant was discarded, the cell count was performed using a Neubauer chamber, and the cell density was adjusted to 1 × 10^6^ cells per tube.

To investigate total cellular protein expression, cell fixation was performed prior to staining. Cells were incubated with 2% (*v*/*v*) paraformaldehyde in PBS buffer for 30 min at room temperature, then washed with 0.1 M glycine in PBS buffer. Subsequently, cells were permeabilized by incubation with 0.01% saponin in PBS for 30 min at room temperature, followed by another wash with PBS buffer. After these steps, to evaluate interleukin-6 (IL-6) expression, cells were incubated with a mouse anti-IL-6 monoclonal primary antibody (Santa Cruz Biotechnology, Dallas, TX, USA) at a 1:100 (*v*/*v*) dilution in PBS buffer containing 1.5% (*w*/*v*) albumin for 1 h at room temperature. Negative control samples were also prepared following the same experimental steps, except for incubation with the primary antibody.

After primary antibody incubation, cells were washed with PBS and centrifuged at 2000 rpm for 5 min; the supernatant was discarded. This was followed by a 40-min incubation protected from light with the Alexa Fluor 594-conjugated secondary antibody (Molecular Probes) at a 1:200 (*v*/*v*) dilution in PBS buffer containing 1.5% (*w*/*v*) albumin. Finally, cells were washed with PBS, centrifuged at 2000 rpm for 5 min at 4 °C, the supernatant discarded, and resuspended in PBS until flow cytometry analysis.

Readings and analyses of IL-6 expression were performed using a FACSCalibur flow cytometer (BD Bioscience, Franklin Lakes, NJ, USA). For each sample, a total of 10,000 events were recorded using CellQuest Pro software (v5.x, BD Bioscience), which sets parameters including cell size (forward scatter—FSC) and granularity/complexity (side scatter—SSC) on a linear scale, followed by channel FL2 on a logarithmic scale, detecting fluorescence emitted by the Alexa Fluor 594-conjugated antibody. Data analysis was performed using Flow Jo vX.0.7 software (Treestar Inc., Ashland, OR, USA). Results were reported as the percentage of positive cells and quantified based on the geometric mean of the tested samples and controls.

### 2.9. Confocal Laser Microscopy Evaluation of IL-6 Expression

Immunofluorescence assays were performed to analyze the IL-6 expression modulated by P_11_-4 before and after cellular stimulation. For this purpose, cell lines were cultured on circular coverslips with an 18 mm diameter, at a density of 1.0 × 10^4^ cells per coverslip, and maintained in 12-well culture plates under standard conditions until reaching 70% confluence. Then, the cells were stimulated by 6.3, 12.6, and 25.2 µmol/L of the P_11_-4 peptide for 6 h. After the stimulation period, the culture medium was carefully removed from the wells, the cells were washed with PBS buffer, and subjected to IL-6 labeling. Cells were incubated for 1 h at 4 °C with a mouse monoclonal anti-IL-6 primary antibody (Santa Cruz Biotechnology, USA), diluted 1:100 (*v*/*v*) in PBS buffer containing 1.5% (*w*/*v*) albumin. Following this incubation, cells were washed with PBS buffer, and the wells were treated with an Alexa Fluor 594-conjugated anti-mouse secondary antibody (Molecular Probes) diluted 1:200 (*v*/*v*) in PBS buffer containing 1% (*w*/*v*) albumin. Cells remained in contact with the antibody for 40 min at 4 °C in the dark.

For nuclear staining, in all conditions mentioned above, cells were incubated with DAPI reagent (4′,6-diamidino-2-phenylindole; Molecular Probes) diluted 1:1000 in PBS with 0.01% saponin for 30 min at room temperature. Upon completion of the procedure, coverslips were washed three times with PBS buffer and mounted onto histological slides using Prolong^®^ Gold Antifade (Invitrogen, Carlsbad, CA, USA). Images were acquired using a confocal laser microscope, Leica SP8 (Leica Microsystems), and analyzed using LAS X software (Leica Microsystems). Representative images from each experimental group were selected for data presentation.

### 2.10. Statistical Analysis

Data were subjected to normality (Shapiro–Wilk) and homoscedasticity (Levene) tests. Data for cell viability was analyzed by ANOVA, two-way complemented by Tukey test was used multiple comparisons. Data for the proliferation assay was analyzed using a nonparametric one-way Kruskal–Wallis test, followed by Dunn’s multiple comparison test with Bonferroni adjustment. In all cases, the global significance level was 5%. For PCR interpretation of pathways and processes associated with the analyzed genes, an over-representation analysis was performed using the clusterProfiler package [7,8]. Enrichment analyses of Kyoto Encyclopedia of Genes and Genomes (KEGG) pathways and Gene Ontology cellular components were conducted. It was considered significant when FDR < 0.05. Data for: cell migration, cytoplasmic calcium concentration, intracellular reactive oxygen species (ROS) production, and interleukin-6 (IL-6) are qualitatively described and presented, not being subjected to statistical analysis.

## 3. Results

### 3.1. Cell Viability

**Figure 1:**
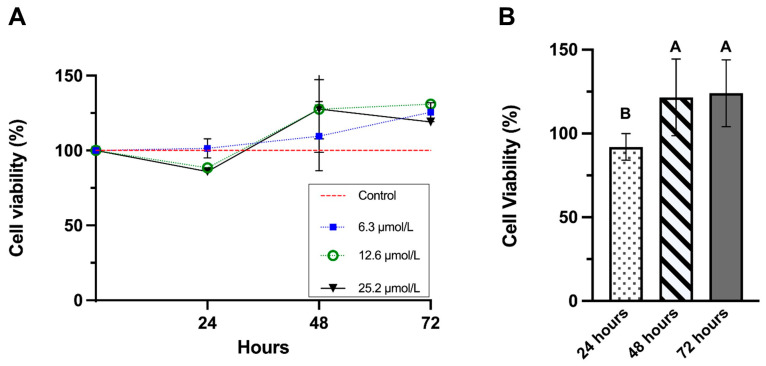
(**A**) Means and standard deviations (*n* = 3) for cell viability (%) of MDPC-23 cells treated with crescent concentrations: 6.3 (blue), 12.6 (green), and 25.2 µmol/L (black) of P11-4 over 72 h. (**B**) Pooled data for significant factor time. Similar uppercase letters indicate a lack of statistically significant differences (ANOVA and Tukey test, *p* > 0.05).

Means and standard deviation for cell viability results for all tested concentrations during the three time points are displayed in Figure 1A. The effects of concentration and exposure time on cell viability were evaluated using a two-way ANOVA, considering two factors: concentration (in three levels: 6.3, 12.6, and 25.2 µmol/L) and time (in three levels: 24, 48, and 72 h).

The two-way ANOVA revealed a significant main effect of time on cell viability (*p* < 0.01, Figure 1B), indicating that viability differed significantly across the exposure periods. Cells exposed for 24 h had significantly lower viability compared to the following periods. These results were expected considering that P_11_-4 concentrations were not statistically significant (*p* > 0.05), indicating that, within the tested range (6.3–25.2 µmol/L), changes in concentration did not significantly impact cell viability when averaged across all time points.

The interaction between concentration and time was also not statistically significant (*p* > 0.05). This result indicates that the pattern of the time effect on cell viability was similar across all concentrations, and that increasing the P_11_-4 concentration did not change the temporal profile of the response.

In summary, cell viability analysis shows that even after 72 h of exposure to P_11_-4 at crescent concentrations remains non-cytotoxic to the MDPC-23 cell line. This evidence shows that the peptide, at the tested concentrations, is not only safe but also poses no risk to the integrity of the dentin-pulp complex.

### 3.2. Cell Proliferation

**Figure 2:**
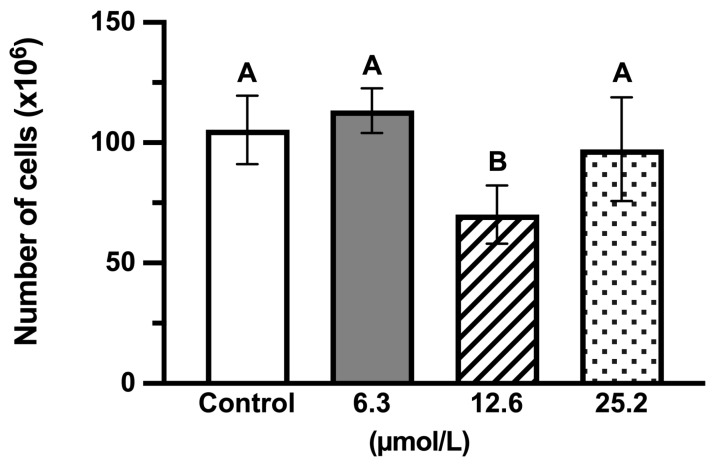
Means and standard deviations (*n* = 3) for cell proliferation of MDPC-23 treated with three concentrations, 6.3, 12.6, and 25.2 µmol/L of P11-4 assessed by DAPI staining. The same letters indicate lack of statistically significant differences (Kruskal–Wallis/Dunn’s test, *p* > 0.05).

The evaluation of cell proliferation by DAPI staining (Figure 2) demonstrated no statistically significant difference in the number of stained nuclei after 24 h of P_11_-4 treatment at concentrations of 6.3 µmol/L and 25.2 µmol/L compared with the control (*p* > 0.05). However, a statistically significant decrease of 29% (*p* < 0.01) was observed at a P_11_-4 concentration of 12.6 µmol/L. The corresponding nuclear density strongly correlates with the results of the cell viability assay, indicating that, overall, P_11_-4 treatment preserved cellular integrity. These findings confirm the potential of this material to maintain a cellular environment favorable for tissue regeneration.

### 3.3. Cell Migration

**Figure 3:**
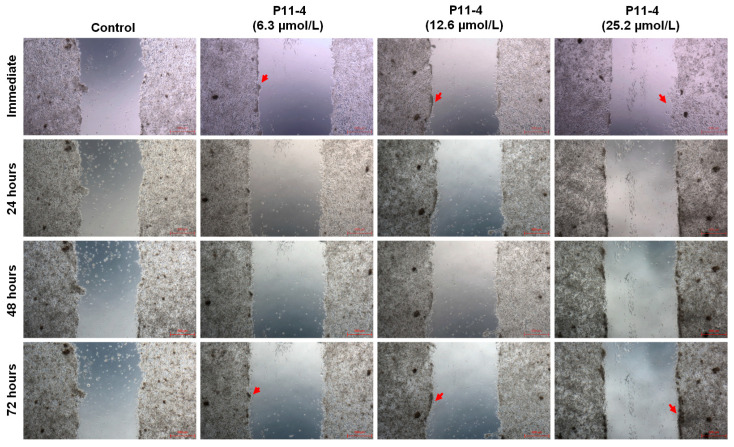
Cell migration of MDPC-23 treated with three concentrations, 6.3, 12.6, and 25.2 µmol/L of P_11_-4 (*n* = 3). The cells were continuously exposed to the P_11_-4 peptide for 72 h, and images from the same field were assessed every 24 h. The red arrows indicate the areas around the wound margins where cell proliferation can be observed.

The wound-healing assay demonstrated that MDPC-23 cells possess a limited migratory capacity. However, as shown in Figure 3, at all tested concentrations, there was an increase in the number of cells within the gap over the evaluated periods, even though complete wound closure was not achieved. More, despite the low migration potential, a significant increase in the confluence of cells located at the edges of the gaps was observed. This finding indicates that the cells’ ability to proliferate remained stable, even in the presence of higher concentrations of P_11_-4.

### 3.4. Cytoplasmic Calcium Concentration

**Figure 4:**
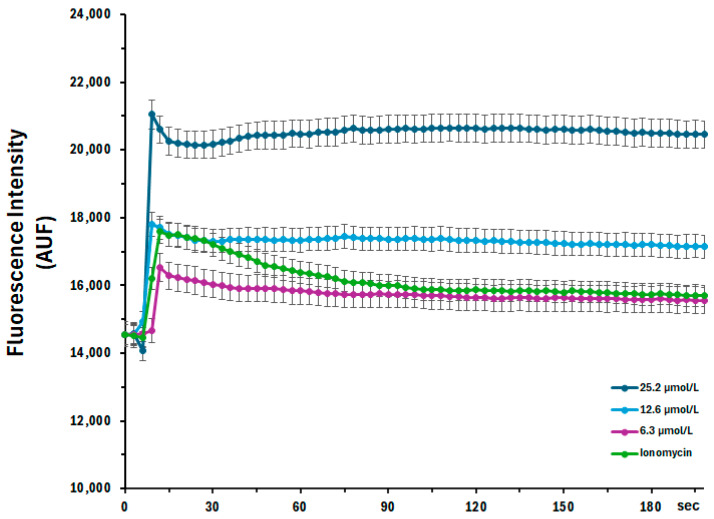
Intracellular calcium influx in MDPC-23 cells treated with 6.3 (pink), 12.6 (light blue), or 25.2 µmol/L (dark blue) of P_11_-4 peptide compared to ionomycin (positive control, green), over 200 s (*n* = 3).

The kinetics of cellular calcium influx mediated by 6.3, 12.6, and 25.2 µmol/L of P_11_-4 peptide, as well as Ionomycin (5 µmol/L), were evaluated over a 200-s interval. A 10-s baseline reading was taken prior to the addition of the peptide in all three experimental groups and in the ionomycin control group. The 25.2 µmol/L concentration showed the highest increase in intracellular calcium levels throughout the assay. The 12.6 µmol/L concentration induced a response similar to that of ionomycin; however, at this P_11_-4 amount, the intracellular calcium concentration remained sustained throughout the experimental period, suggesting the activation of the store-operated calcium channels (SOCE). As expected, the concentration of 6.3 µmol/L practically did not promote cellular calcium influx, suggesting a dose-dependent relationship between the P_11_-4 concentration and the potential damage to cellular metabolism (Figure 4).

### 3.5. Intracellular Reactive Oxygen Species (ROS) Production

ROS are players in cellular signaling and the stress response. The specific levels and types of ROS present can determine a cell’s capacity to undergo cell death.

**Figure 5:**
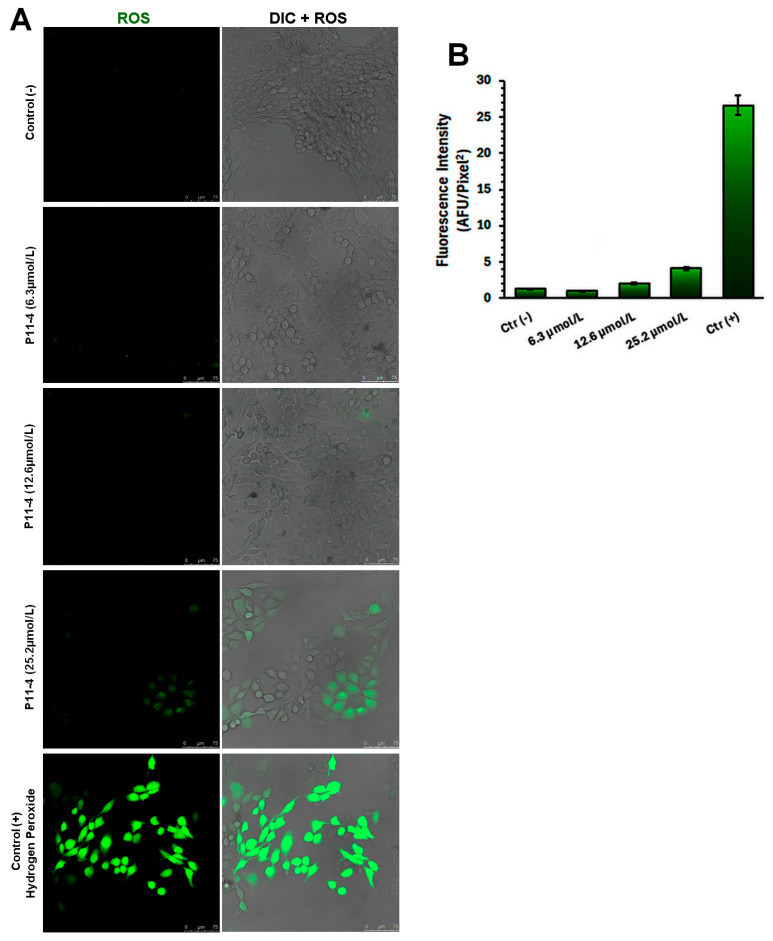
(**A**) Representative confocal laser scanning microscopy images illustrating intracellular reactive oxygen species (ROS) generation in MDPC-23 cells following exposure to P_11_-4 at concentrations of 6.3, 12.6, and 25.2 µmol/L. ROS formation was detected using the redox-sensitive fluorescent probe H_2_DCFDA, which emits green fluorescence upon intracellular oxidation. For each experimental condition, green-channel images depicting ROS-related fluorescence are shown on the left, while merged images combining ROS fluorescence with differential interference contrast (DIC) are presented on the right (ROS + DIC), allowing visualization of oxidative activity in relation to cellular morphology. Negative control (−) corresponds to the absence of P_11_-4, while the positive control (+) consists of cells treated with hydrogen peroxide (100 µmol/L). (**B**) Quantitative analysis of ROS-associated fluorescence intensity, expressed as mean fluorescence emission, enabling comparison of intracellular oxidative levels among experimental groups. Scale bar: 75 µm.

Figure 5 demonstrates that only the 25.2 μmol/L concentration of P_11_-4 induced visible intracellular fluorescence, indicating an increase in ROS production. In contrast, the lower concentrations of 6.3 μmol/L and 12.6 μmol/L, along with the negative control group, did not show any detectable fluorescent signal, suggesting that there was no significant oxidative stress mediated by P_11_-4 under these conditions.

### 3.6. P_11_-4 Modulating Gene Expression in MDPC-23 Cells

The PCR Array is a tool designed to evaluate the expression of specific gene panels under various biological conditions using real-time PCR. The osteogenesis-related gene array includes a list of pathway-specific genes along with five housekeeping genes.

**Figure 6:**
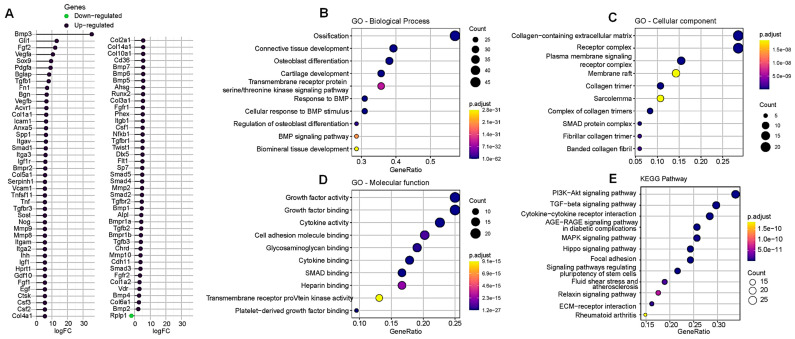
Gene Expression Profiling and Enrichment Analysis of the Target Genes. (**A**) Lollipop plot showing differential gene expression between the P_11_-4 group and the control. The subsequent panels display the enrichment analysis performed on the resulting gene set. The plots are ranked by Gene Ratio, with point size indicating the number of genes associated with each term, and color representing the *p*-value. (**B**) Gene Ontology (GO)—Biological Process; (**C**) GO—Cellular Component; (**D**) GO—Molecular Function; (**E**) KEGG pathway enrichment analysis.

In this study, 84 genes were profiled in two samples: one that served as the control without P_11_-4 treatment and another that consisted of cells induced for 24 h with 6.3 μmol/L of the peptide. Figure 6 demonstrates that P_11_-4 positively modulates 79 genes directly involved in mineral deposition. Additionally, three genes show no change in expression, while only one gene is subjected to negative regulation. This pattern highlights the significant impact of P_11_-4 on mineral-related genetic functions and confirms its role in this biological process.

In addition to identifying specific gene modulation (Figure 6A), it was possible to correlate the expression of these genes to several factors, including biological processes (Figure 6B), cellular components (Figure 6C), molecular functions (Figure 6D), and the associated cell signaling pathways (Figure 6E).

Regarding biological processes, the results indicate that P_11_-4 significantly enhances the response to bone morphogenetic protein (BMP), evidenced by a 35.48-fold upregulation of the BMP-3 gene. This upregulation also includes the cellular response to BMP stimulation and the BMP signaling pathway. Additionally, we observed positive modulation of genes associated with tissue mineralization and the differentiation of cells that secrete mineralized matrix.

The modulation of cellular components shows that, in nearly all significant correlations, there is an increase in the collagen expression in its trimeric form, whether it is individual or complexed. Additionally, the data shows an increase on extracellular matrix collagen as the strongest correlation point. Furthermore, the expression of the SMAD protein complex also emerges as an important modulated gene. Analysis of molecular functions shows that clustering, as expected, highlights the positive regulation of growth factors, cytokine activity, cell-adhesion molecule binding, and glycosaminoglycan binding as the most prominent features associated with P_11_-4–induced modulation. Interestingly, SMAD binding also emerged as a highly correlated functional category in this analysis.

Finally, the analysis of the most prominent signaling pathways, based on the overexpressed genes, clearly indicates that the PI3K and TGF-β pathways were significantly impacted by the pro-mineralizing effects of the P_11_-4 peptide treatment. These results complement those previously shown, as the PI3K signaling pathway is crucial for the remodeling process of mineralized matrices, influencing the proliferation and differentiation of specific tissue cells. Meanwhile, the TGF-β pathway, which is mediated by SMAD, also plays a role in the mineralization process.

### 3.7. Interleukin-6 (IL-6) Expression

IL-6 is a pleiotropic cytokine with diverse biological and clinical functions, particularly responsible for inducing the synthesis of most acute-phase proteins associated with inflammation [9].

**Figure 7:**
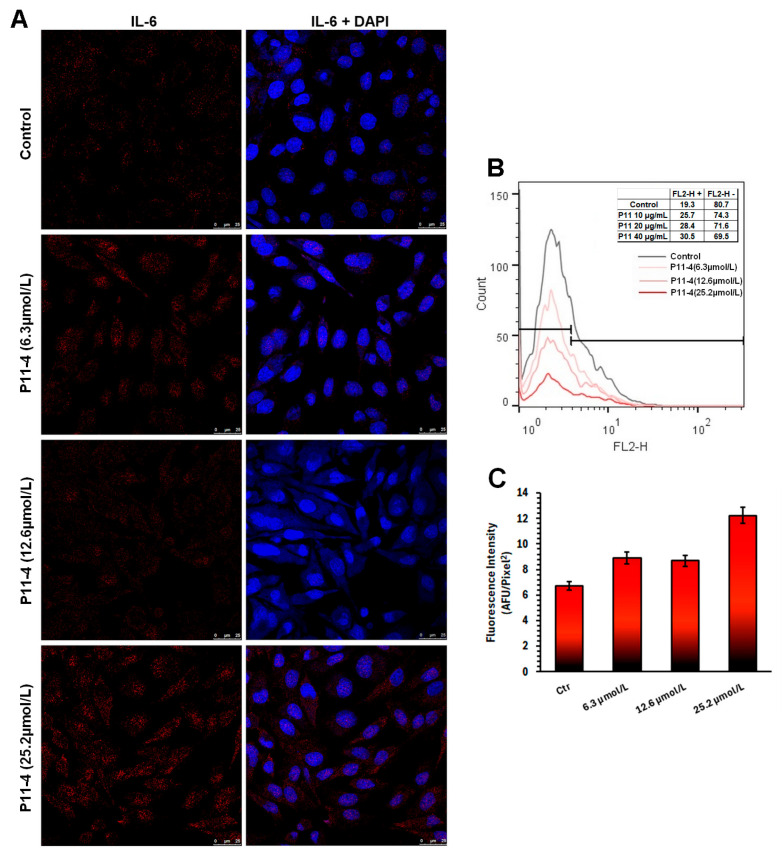
(**A**) Representative confocal laser scanning microscopy images of intracellular interleukin-6 (IL-6) expression in MDPC-23 cells following exposure to the P_11_-4 at concentrations of 6.3, 12.6, and 25.2 µmol/L. IL-6 was detected by immunofluorescence labeling, with the corresponding fluorescence signal acquired in the red channel (left). Cell nuclei were counterstained with DAPI and visualized in the blue channel (right). (**B**) Flow cytometric analysis of IL-6 expression in MDPC-23 cells under the same experimental conditions, providing quantitative single-cell–level assessment of cytokine expression. (**C**) Quantitative analysis of IL-6 immunofluorescence intensity, expressed as mean fluorescence emission, allowing comparison of intracellular IL-6 levels among experimental groups. Scale bar: 25 µm.

The assessment of IL-6 protein expression by confocal laser scanning microscopy (Figure 7A) and flow cytometry (Figure 7B) revealed complementary findings, indicating a modest increase in IL-6 levels in cells stimulated with 25.2 µmol/L of P_11_-4. The results show that even at higher concentrations, P_11_-4 was unable to cause sufficient cellular changes to activate the expression of this specific cytokine.

## 4. Discussion

Dentin mineralization is a biphasic process that begins with the synthesis and deposition of the extracellular matrix (ECM) by odontoblasts, followed by the subsequent mineralization of that matrix. The ECM is primarily composed of type I collagen (about 90%), although types III and V collagen have also been identified in dentin [10]. While NCPs make up a smaller portion of the dentin organic matrix, they play essential regulatory roles during the mineralization process.

Odontoblasts are long-lived, post-mitotic cells located along the dentin-pulp interface. Like neurons and cardiomyocytes, odontoblasts are largely stable and do not undergo replacement once they have fully differentiated [11]. Mature odontoblasts contain extensive rough and smooth endoplasmic reticulum, a well-developed Golgi apparatus, and abundant mitochondria, which allow for the synthesis of a wide variety of proteins involved in dentin mineralization [12].

Our research group has focused on understanding the mechanisms by which the P_11_-4 peptide enhances and guides dentin mineralization. Recent findings indicate that P_11_-4 not only protects collagen from proteolytic degradation [6] but also facilitates calcium deposition at the C-terminal region of the collagen telopeptide [2]. Despite these significant advances, there are still important knowledge gaps regarding how dentin-forming cells respond to P_11_-4 stimulation. Therefore, the present study is the first to evaluate the potential cytotoxic effects of P_11_-4 on odontoblasts and to characterize the cellular responses triggered by this self-assembled peptide in establishing a favorable microenvironment for dentin remineralization.

The MTT cell viability assay showed that, despite presenting a slight reduction in viability observed during the first 24 h for the highest concentrations, P_11_-4 did not induce cytotoxic responses in MDPC-23 cells up to 72 h of exposure. In this context, precise assessment of cell proliferation rates in in vitro studies is essential for apoptosis evaluations and constitutes a critical initial step in assays that help to confirm early-stage toxicity [13]. Consistent with the viability data, our findings demonstrated that 12.6 μmol/L of P_11_-4 caused a modest decrease in cell proliferation during the first 24 h of incubation. Conversely, the lowest concentration tested promoted a slight increase in cell division over the evaluated period. Taken together, these results indicate that P_11_-4 does not induce alterations capable of compromising the cell cycle.

Cell locomotion is also widely investigated in vitro to elucidate its fundamental molecular mechanisms, characterize the signaling pathways involved, and support the identification of potential therapeutic targets [14]. Our findings indicate that, despite the inherently low migratory capacity of MDPC-23 cells, treatment with P_11_-4 did not elicit any detectable changes in their migratory behavior. However, while our data demonstrate that P_11_-4 does not significantly alter cellular behavior, we also assessed the potential harmful metabolic changes caused by the P_11_-4 peptide. Increases in cytosolic Ca^2+^ ions are considered a crucial signal in regulating various cellular processes, including cell injury, cell death, and cell differentiation. The intracellular calcium influx assay demonstrates that only the highest concentration tested was able to induce sustained Ca^2+^ influx. This cellular behavior in response to exogenous stimuli is primarily mediated by store-operated calcium channels (SOCs) [15]. These channels are widely expressed in excitatory and non-excitatory cells, where they mediate significant store-operated calcium entry (SOCE), a process implicated in numerous biological functions [16], including triggering cell death by activating degradative enzymes and disrupting mitochondrial function [17]. Following the rationale, reactive oxygen species (ROS) also play several essential roles in cellular behavior; dysregulated ROS production can lead to cell death through oxidative stress or cytosolic calcium imbalance [18]. The intracellular ROS release data are consistent with the calcium influx findings, indicating that only the highest concentration of P_11_-4 was able to induce a modest increase in both cytosolic calcium and intracellular ROS levels. These results demonstrate that the commonly used concentrations of the peptide are not sufficient to trigger pathways associated with cell damage. Thus, based on the findings regarding cell viability, cell proliferation, ROS production, and calcium influx, the first null hypothesis can be accepted.

On the other hand, although P_11_-4 did not induce metabolic damage in the cells, it demonstrated a strong capacity to modulate the expression of key genes involved in extracellular matrix mineralization. Therefore, the second null hypothesis can be rejected. Among all the evaluated genes, BMP3, FGF2, VEGFA, and Sox9 presented the highest levels of upregulation. Interestingly, BMP3 has traditionally been characterized as an antagonist of BMP2 during osteogenesis. Mechanistic studies now suggest that this antagonism arises from competitive interactions for shared intracellular signaling components between the TGF-β/activin and BMP pathways, thereby modulating downstream SMAD activation profiles. Moreover, BMP3 is the most abundant BMP detected in demineralized bone, underscoring its potential role as a key regulator of osteogenic BMP signaling dynamics in vivo [19]. The coordination of angiogenesis and osteogenesis is governed by tightly regulated signaling networks, among which the VEGF and FGF pathways play central and interconnected roles. The VEGF signaling pathway is essential for coupling vascular and bone formation processes. Through paracrine binding to its receptor, VEGFR, VEGF initiates intracellular signaling cascades that include the PI3K/Akt, PKC/MAPK, and p38/MAPK pathways, which promote endothelial cell proliferation, migration, and survival while simultaneously enhancing the recruitment and differentiation of osteoprogenitor cells [20]. In parallel, the FGF signaling axis also regulates key events in osteogenesis and angiogenesis [21]. FGFs exert their effects by binding to FGFRs, a family of tyrosine kinase receptors that activate multiple downstream signaling events. Among these ligands, FGF9 is particularly important during long bone repair, as it facilitates both vascularization and bone formation [22]. Interestingly, FGF9’s pro-angiogenic effects are mediated in part through the regulation of VEGFA expression, which was considerably upregulated in our study, highlighting a functional interplay between the FGF and VEGF pathways in orchestrating efficient bone regeneration.

Sox9 is a key transcription factor that drives the expression of chondrocyte-specific genes such as Col2α1 and Col11α1, and it is essential for the sequential stages of chondrocyte differentiation and cartilage formation [23]. The best-characterized mechanism involving Sox9 during osteogenesis is its direct interaction with RUNX2, a transcription factor required for osteoblast differentiation and chondrocyte maturation both in vivo and in vitro [24]. Interestingly, RUNX2 expression was also upregulated after P_11_-4 stimulation in our study. Although the full range of mechanisms by which Sox9 contributes to ossification remains under investigation, evidence indicates that Sox9 can directly repress VEGFA transcription by binding to SRY recognition sites within the VEGFA gene [25].

In accordance with these mechanistic insights, our findings demonstrate that P_11_-4 positively modulates multiple molecular pathways associated with extracellular matrix mineralization. In addition to the upregulation of Sox9 and RUNX2-related targets, genes involved in matrix organization and chondrogenic/osteogenic processes, such as Col2α1, Col4α1, Col14α1, and Col10α1, were significantly overexpressed. Furthermore, other pathways previously discussed, including those related to SMAD signaling and MMPs activity, were also positively modulated, reinforcing the role of P_11_-4 in promoting a molecular microenvironment favorable to mineralized tissue formation.

A lipid kinase, phosphoinositide 3-kinases (PI3K), is frequently associated with increases in tyrosine phosphorylation triggered by growth and differentiation factors in dental pulp cells [26]. In osteogenic cells, BMP2 stimulates both tyrosine phosphorylation and PI3K activation, establishing a signaling configuration in which PI3K and its downstream effector, the serine/threonine kinase Akt, are required to drive BMP2-induced osteoblast differentiation [27]. Evidence further indicates a bidirectional cross-talk between BMP-specific SMAD proteins and the PI3K/Akt axis, where PI3K/Akt signaling potentiates SMAD-dependent transcription of BMP2. This integrated signaling circuitry is essential for sustaining the proliferative and differentiation programs of osteogenic cells [28].

The gene expression analysis reveals a robust association between P_11_-4 induced cells and PI3K pathway activation. In line with recent literature, the second-highest association is observed with the TGF-β signaling pathway. This pathway can be regulated by PI3K under certain biological conditions, such as epithelial–mesenchymal transition and matrix protein expansion [29].

In contrast, the only gene that exhibited downregulation was Ribosomal Protein Lateral Stalk Subunit P1 (RPLP1), a gene typically listed among the most upregulated markers associated with osteosarcoma metastasis [30]. Its reduced expression in normal cells following P_11_-4 stimulation suggests that RPLP1 is unlikely to play a significant role in physiological bone formation, reinforcing the notion that its upregulation is more closely linked to pathological, rather than healthy, osteogenic processes. In this context, elevated IL-6 expression correlates with aggressiveness and tumor staging in osteosarcomas. However, our findings indicate that the P_11_-4 peptide stimulation did not enhance cellular IL-6 expression.

We have previously demonstrated that the P_11_-4 peptide, in its β-sheet-rich polymeric form, can electrostatically bind to specific regions of type I collagen. This binding reinforces the collagen structure and protects it from proteolytic degradation by providing steric inhibition [6]. Our earlier work also showed that collagen-bound P_11_-4 promotes the guided nucleation of hydroxyapatite crystals and accelerates tissue remineralization [2].

Given that predentin cells are intimately involved in the remineralization of both healthy and pathological dentin, presenting a possible role of P_11_-4 in cell signaling and protein expression in odontoblast-like cells, represents a logical and necessary research progression. Here, we provide the first evidence suggesting that the self-assembling peptide P_11_-4 can modulate cellular metabolism and regulate the expression of genes associated with dentin mineralization. Moreover, our data indicate that P_11_-4 does not induce apoptosis or necrosis, nor does it abnormally enhance cell proliferation or migration, even at high concentrations, supporting its profile as a biocompatible and non-toxic biomaterial.

## 5. Conclusions

This study provides the first comprehensive evidence that P_11_-4 not only interacts structurally with dentin matrix components but also modulates key cellular and molecular pathways involved in odontoblast-mediated mineralization. P_11_-4 demonstrated a favorable biocompatibility profile, as it did not induce cytotoxicity. In contrast, P_11_-4 significantly upregulated a broad set of genes associated with extracellular matrix organization, osteogenic differentiation, and mineralization-regulatory transcription factors such as Sox9 and RUNX2. Pathway enrichment analyses further highlighted strong activation of PI3K- and TGF-β-related signaling networks, which are essential for osteogenic commitment and coordinated matrix mineralization. Taken together, P_11_-4 emerges as a safe and biologically potent biomimetic peptide with considerable promise for advancing regenerative strategies and promoting targeted dentin remineralization.

## Data Availability

The original contributions presented in the study are included in the article, further inquiries can be directed to the corresponding author.
